# Rural Computed Tomography - a model for access to rapid stroke care in sparsely populated areas?

**DOI:** 10.1186/s12913-026-14307-6

**Published:** 2026-03-19

**Authors:** Jørgen Ibsen, Maren Ranhoff Hov, Jessalyn Kathryn Holodinsky, Per Olav Kaasa, Are Hugo Pripp, Christian Georg Lund, Christian Hall

**Affiliations:** 1 Ringerike Hospital, Vestre Viken Hospital Trust, Box 800, Drammen, 3004 Norway; 2 Faculty of Medicine, University of Oslo, Oslo, Norway; 3 Department of Neurology, Oslo University Hospital, Oslo, Norway; 4 Oslo Metropolitan University, Oslo, Norway; 5 The Norwegian Air Ambulance Foundation, Oslo, Norway; 6 Department of Emergency Medicine, Community Health sciences and Clinical Neurosciences, Centre for Health Informatics, O`Brien Inst, for Public Health, Hotchkiss Brain Inst, Cumming School of Medicine, University of Calgary, Calgary, Canada; 7 Norwegian Mapping Authority, Honefoss, Norway; 8 Oslo Centre of Biostatistics and Epidemiology, Research Support Services, Oslo University Hospital, Oslo, Norway; 9 Institute of Clinical Medicine, University of Oslo, Oslo, Norway

**Keywords:** Stroke, Computed tomography, Thrombolysis, Prehospital, Rural

## Abstract

**Background:**

Patients living in rural districts may have limited access to acute stroke treatment due to long transport times to hospital facilities with radiology and revascularization therapy. This study simulates the utility of transferring acute diagnostics and treatment into rural areas by deployment of rural Computed Tomography (CT) services.

**Methods:**

In Hallingdal county, Norway, a CT and thrombolytic station is operated in a local medical centre. We hypothetically deployed similar stations in rural Norway to explore the impact of such model of prehospital rural acute stroke treatment on ambulance transport times and patient outcomes after ischemic stroke. In addition, we described consequent changes in transport strategy for patients with suspected Large Vessel Occlusion (LVO) stroke.

**Results:**

Significant reductions in hypothetical transport times were obtained after deployment of 30 rural CT stations. The part of the Norwegian population not eligible for thrombolysis within the recommended time window due to long transport time decreased from 11% to 1% (*p*< 0.001). By simulation of 1000 ischemic strokes, the estimated odds ratio for good outcome judged by modified Rankin Scale after thrombolysis for the rural population increased from 1.37 to 1.61 (*p* < 0.001). When LVO stroke was suspected, rural CT stations slightly increased the geographical area in which patients would receive local thrombolysis before transport to endovascular treatment.

**Conclusions:**

Deployment of rural CT stations may facilitate rapid triage and early thrombolysis for patients with acute ischemic stroke living furthest away from hospital and this concept may be applicable to other health care systems worldwide. However, further research is needed to confirm its utility in the clinical setting.

**Supplementary Information:**

The online version contains supplementary material available at 10.1186/s12913-026-14307-6.

## Introduction

The effect of thrombolytic treatment for ischemic stroke is highly dependent on time [1, 2]. Intravenous thrombolysis with alteplase is approved for administration within 4.5 h from onset of stroke symptoms to treatment (OTT) in Norway [3–5]. However, this time window may be extended up to 9 h for selected patients [6]. For Large Vessel Occlusion (LVO) stroke, Endovascular Treatment (EVT) is the preferred method of revascularisation, frequently after initial thrombolysis [7–11]. A Computed Tomography (CT) scanning of the brain including angiography must be performed as early as possible to exclude intracerebral hemorrhage (ICH) and decide treatment strategy.

The Norwegian Health Authorities aim to offer equal health services for all inhabitants [12]. However, the time dependent effect of thrombolytic treatment constitutes a public health challenge as it introduces an inequality in the access to effective treatment. CT equipment is traditionally hospital based and operated by radiographers. Thus, patients living in rural areas are disadvantaged as the effective therapeutic window is partly consumed by the time used during transportation to hospital [13, 14]. To reduce time lost in transportation, prehospital treatment models as in Mobile Stroke Units (MSUs) have been implemented in different research settings and tested in a semi-urban area of Southern Norway [15–17]. The rural population is underserved in terms of emergency stroke treatment and further research is needed to solve this problem.

Vestre Viken Hospital Trust, Norway has developed a de novo concept consisting of a prehospital CT and thrombolytic service located in the rural district of Hallingdal [18].

The service is during daytime delivered by a local radiographer, a nurse and a general practitioner being supervised by telestroke from a physician at the nearest Primary Stroke Centre (PSC). Outside office hours, the CT scanner is remotely controlled by a radiographer from the PSC while trained local paramedics assist in the CT examination, perform a neurological exam by National Institute of Health Stroke Scale (NIHSS) and, if advised from the PSC physician, administer thrombolytic therapy (alteplase). 25 local paramedics are trained for this task, and skills are maintained by monthly exercise and education. Outside office hours no CTA is available, however clinically suspected LVO stroke (NIHSS > 6, exclusion of haemorrhage) is preferred for direct transport to Comprehensive Stroke Centre (CSC). Transport time from the rural CT station to the PSC is 98 min by ambulance car, to CSC 144 min (38 min by helicopter, The Norwegian Air Ambulance Foundation, unpublished).

In the present study, the primary objective was to explore the potential effects of a national roll out of rural CT stations for early stroke triage and thrombolysis to all parts of Norway. Hypothetically, we deployed multiple CT stations nationwide and analysed by simulation the potential effects on transport time, the consequent changes in OTT and estimated odds ratio (OR) for good outcome after thrombolysis. In addition, we explored to what extent such decentralized diagnostics and thrombolysis would influence on national transport strategy for patients with suspected LVO stroke.

## Methods

Norway consists of large areas of forest and mountains and has the second lowest population density (18/km^2^) in Europe [19]. The country has a long coastline with deep fjords characterizing the coastal infrastructure. The road standard is of variable quality sometimes resulting in long patient transport times by ambulance car, not seldom in challenging weather conditions [20]. According to Statistics Norway, 18% of the Norwegian population live in areas defined as rural with an aging of the population compared to urban areas [21]. The population density in especially Northern Norway is low, but the relative incidence of stroke in these areas is high [22]. Patients with acute stroke symptoms are encouraged to alarm the emergency medical system. Most frequently, a car ambulance staffed with two paramedics transports the patient to the nearest hospital. According to the Norwegian Stroke Registry (NSR), only 4% of stroke patients are transported by the helicopter emergency medical service (HEMS) (Norwegian Stroke Registry, unpublished data).

### Estimation of time components from onset of symptoms to treatment

Transport time is a substantial part of the total OTT. In our analyses, we entered reported data for the other time components from the NSR and the Norwegian Health Quality Database [22, 23]. The time from onset of symptoms to alarm included all patients with a final diagnosis of stroke. Due to lack of Norwegian registry data, ambulance on scene time was set as reported in a Danish study [24]. According to our estimates, 110 min of OTT is not due to transport, Supplementary Table 1.

### Calculation of transport times

Utilizing ArcGIS Desktop (ArcMap 10.4.1) software, we applied a total of 221,551 geographical polygons of 250 m^2^ across Norway, Supplementary Fig. 1. In total these circumscribed the homes of 5,274,338 individuals. 6,884 individuals living at locations without road access were not included in the analysis. Road infrastructure, including one-way roads, road quality and use of ferries were obtained from The National Road Database [20].

Applying the Closest-Facility tool (ArcGIS Network Analyst), we calculated transport times in ambulance to the nearest of the 48 Norwegian hospitals offering acute CT diagnostics and intravenous thrombolytic therapy for patients with stroke. Transport time in ambulance for stroke patients was set to 80% of the calculated driving time at speed limits [25].

The total OTT was finally calculated as the sum of the median time components.

### Effects of CT deployment

The hypothetical effects of rural CT deployment were analyzed in ArcGIS by applying an iterative computational process in which possible new CT locations were selected from a Geodata base of 3,000 public buildings with road access (N50, Norwegian Mapping Authority). We deployed 10, 20 and 30 CT stations by the Location Allocation and Maximize. Attendance tools in ArcGIS. We restricted deployment to rural areas with current ambulance transport times > 70 min aiming to obtain transport time reduction for the population in these selected areas. The software selected CT locations for maximum reduction of transport time. More densely populated areas were favoured in the CT location process aiming to maximize the total reduction of transport times. The time constraint was selected aiming to increase the part of the population achieving a total OTT < 180 min (110 min not in transport), resulting in a possible highly effective thrombolysis [2]. In addition, we analyzed the influence of CT deployment on the part of the population with a total OTT > 270 min, presently denying them thrombolytic treatment. The potential clinical effect of CT deployment on outcome after thrombolysis was calculated by entering the estimated OTT for the different CT deployment alternatives into a formula derived from the Emberson et al. meta-analysis relating time to thrombolysis to OR for good outcome (modified Ranking Scale (mRS) 0–1) after 3 months (OR = exp (0.2749 − 0.1458 [t – 4.02] / 1.228) [2]. For the group of individuals with an OTT > 270 min OR were set to 1 (no thrombolysis given).

### Influence of CT deployment on transport strategy in patients with suspected LVO stroke

The consequences of hypothetical rural CT deployment for transport routing when LVO stroke was suspected were analyzed by using software from DESTINE Health (Calgary, Canada) based upon the transport model developed by Holodinsky et al. [26]. The software illustrates the influence of driving distance to hospital on two alternative transport strategies for revascularisation therapy: “drip and ship” (local thrombolysis prior to transfer to an EVT centre) vs. “mothership” (direct transport to EVT centre). DESTINE Health’s software was customized for the Norwegian stroke system including the 48 Norwegian hospitals receiving patients with acute stroke, among these seven hospitals providing EVT. According to the model, suspected stroke was prehospitally identified by the FAST mnemonic (Facial drooping, Arm weakness, Speech difficulties, Time). We considered patients exhibiting any symptoms on the screening tool to be a suspected stroke patient (potential candidate for treatment). Among FAST+ patients we assumed 14.5% to be experiencing an LVO and 31% to be experiencing an ischemic stroke not due to LVO [26]. Time parameters were according to calculated estimates, Supplementary Table 1. Door to needle times for each hospital were obtained from NHR [22]. Door to groin puncture times were directly reported from the seven centres offering EVT. Time for needle to door out at local thrombolysis centre is not registered in NSR but was set to 30 min. Based on data from our rural CT study, door in to door out time at rural CT stations was estimated to 50 min [18]. After deploying the rural CT stations, their locations were transferred to the DESTINE Health software and entered into the analysis.

### Statistical analysis

To explore the potential clinical benefit of estimated changes in time to thrombolysis we ran a Monte Carlo simulation of 1,000 ischemic strokes under each scenario using the software STATA 17. The simulation was set to represent a realistic number of thrombolytic treatments over a 5-year period for the rural subgroup with an incidence of ischemic stroke of 200/100,000 and a frequency of thrombolysis of 25%. The simulation results were applied in an analysis of changes in access to thrombolysis as well as estimated changes in OR for good outcome. Statistical gamma distributions with selected shape α and scale β parameters were explored as simulation models for activity duration, Supplementary Table 2. They were numerically validated by comparing median, interquartile range, minimum and maximum time values and compared to realistic real-world patient data.

## Results

### Access to thrombolysis

**Fig. 1:**
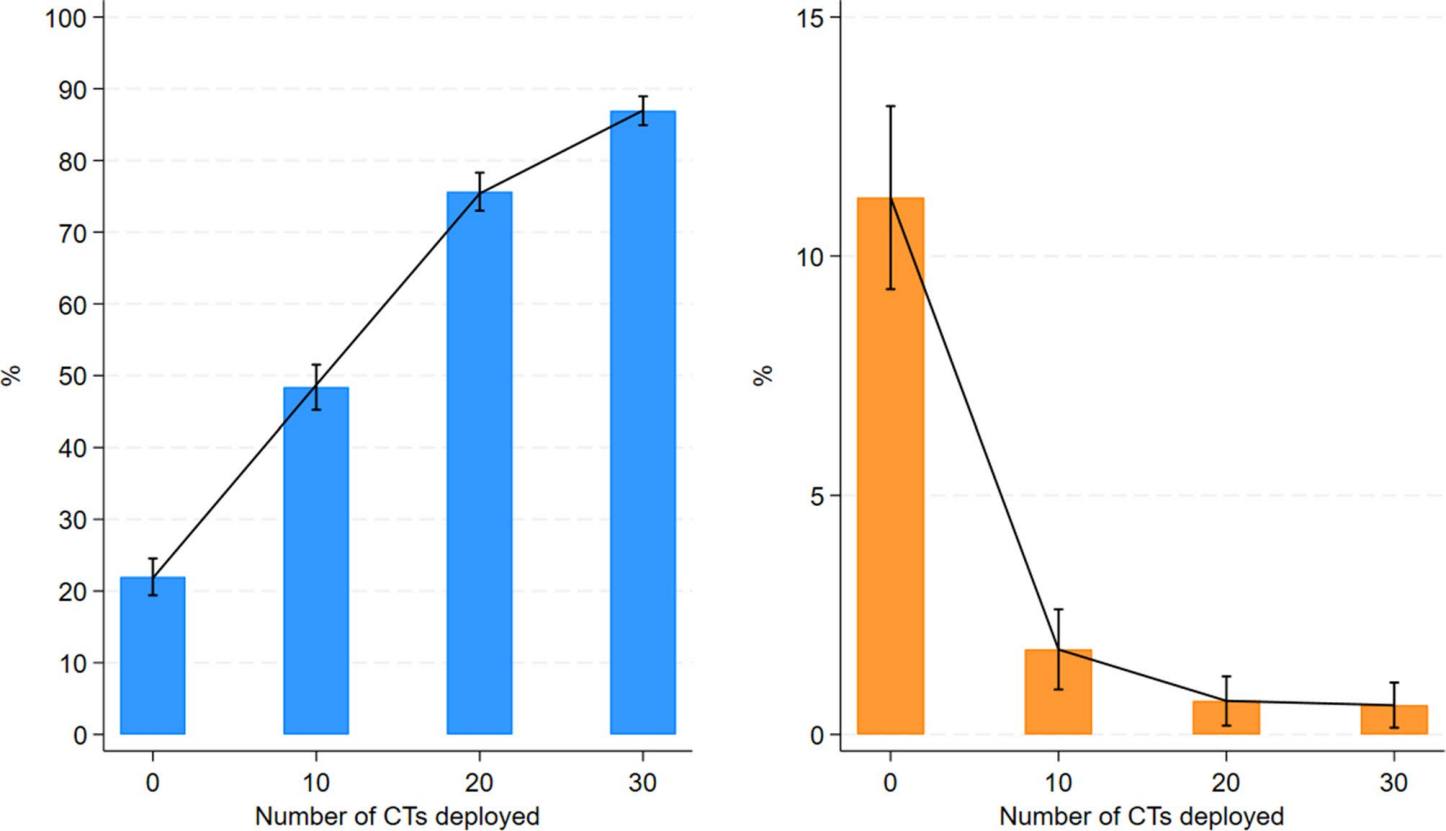
Simulation of 1000 strokes. Graphs illustrating the percentages (mean, 95% CI) of the rural population with OTT < 180 min (blue bars, left) and OTT > 270 min (orange bars, right) after CT deployment

In total 437,687 individuals (8% of the population) had a home location with an estimated ambulance transport time to hospital > 70 min. For these individuals, deployment of 10, 20 and 30 CT stations reduced median (IQR) transport time to 74 (31–105), 48 (15–82) and 31 (9–72) minutes respectively, Supplementary Table 1. CT deployment was restricted to areas with ambulance transport time > 70 min. The Monte Carlo simulation resulted in 22% (*n* = 96,291) of the population potentially achieving a total OTT < 180 min in the existing situation. After CT deployment this proportion increased to 87% (*n* = 380,788). Similarly, the proportion of individuals denied access to thrombolysis (OTT > 270 min) decreased from 11% (*n* = 48,146) to 1% (*n* = 4,367), Fig. 1.

### Impact on OTT and odds ratio for good outcome after thrombolysis

**Fig. 2:**
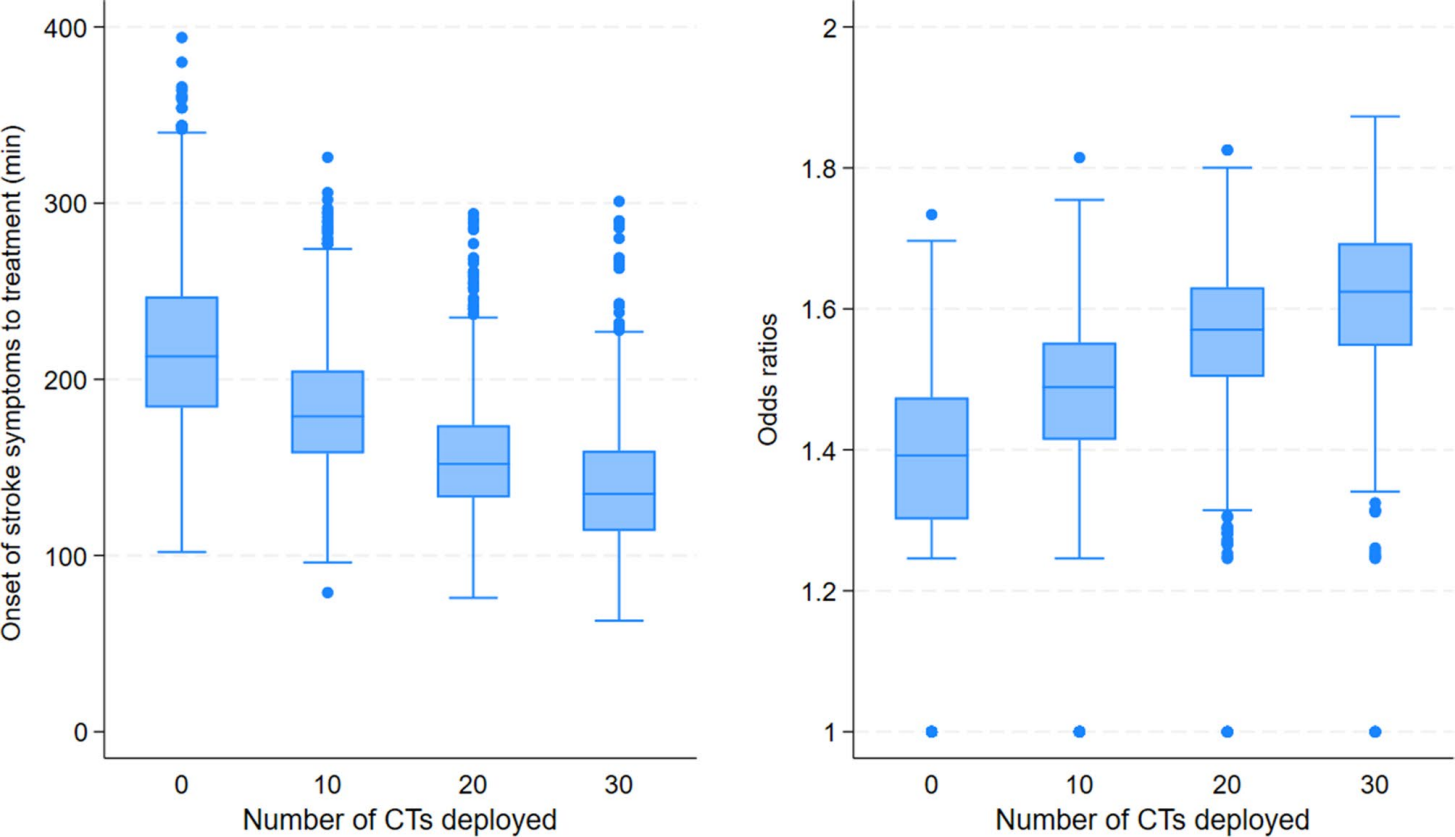
Simulation of estimated effects in median (IQR, min/max) of deployment of 10, 20 and 30 CT stations for the rural subgroup. The left boxplot illustrates the effect on OTT and the right illustrates the effect on OR for good outcome (mRS 0–1) after thrombolysis

After deployment of 10, 20 and 30 CT stations in the rural areas, the simulation of 1,000 thrombolytic treatments of ischemic strokes showed a reduction in mean OTT from 215 min to 184, 158 and 141 min respectively. In addition, we observed an increase in the mean OR for good outcome (mRS 0–1) from 1.37 to 1.47, 1.55 and to 1.61, Fig. 2.

### Geographical location and transport strategy

**Fig. 3:**
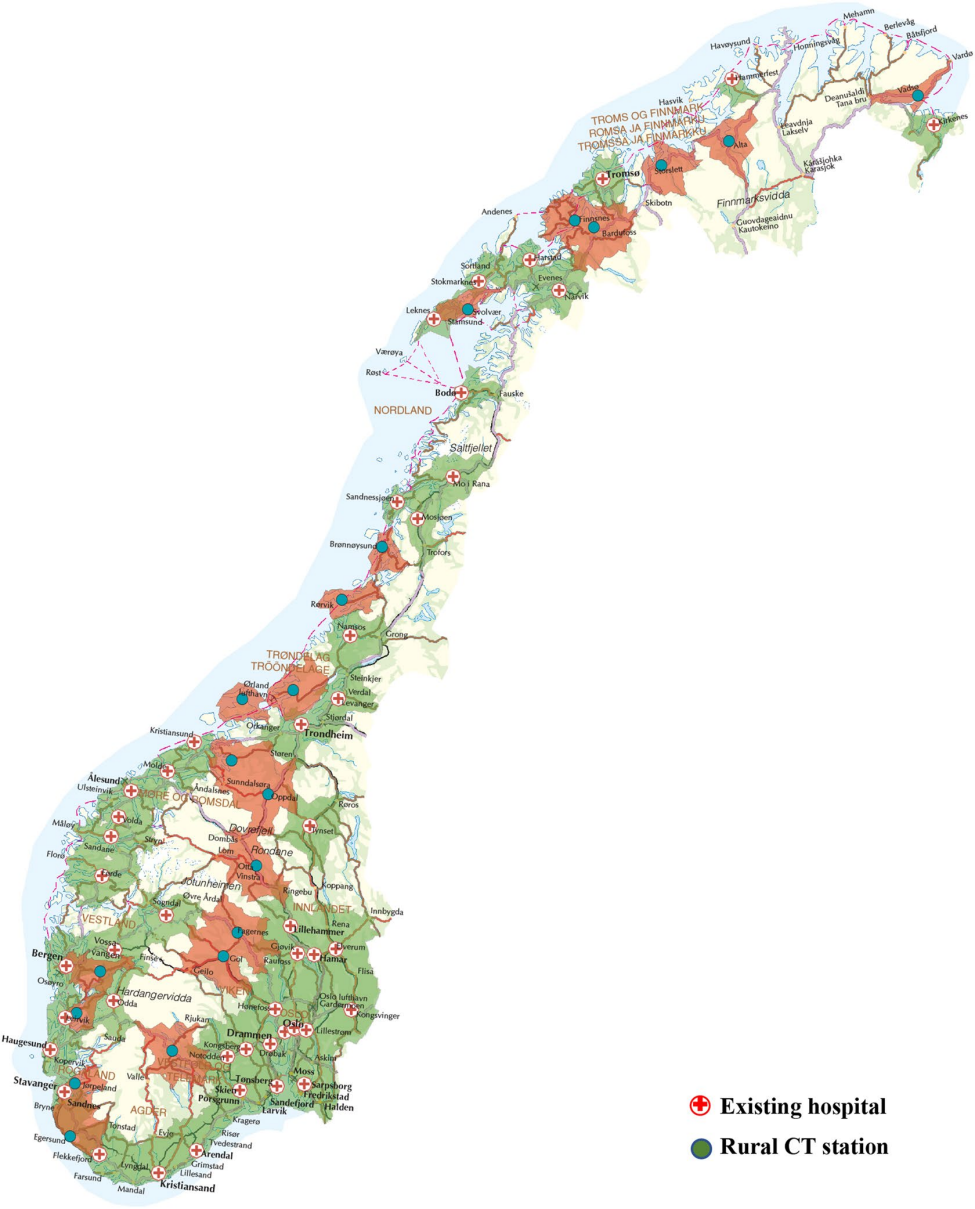
The deployment of 20 CT stations for the rural subgroup. Green color depicts areas in which the population live < 70 min of ambulance transport time from an existing hospital. Red color depicts catchment areas for the deployed CT stations with ambulance transport time < 70 min

The geographical deployment of CT stations in the rural subgroup area is presented in Fig. 3. As an example, we present the existing situation and deployment of 20 stations. The deployment of 10 and 30 CT stations is presented in Supplementary Figs. 2 and 3. When deploying 20 stations, the software co-located nine to already existing District Medical Centres.

**Fig. 4:**
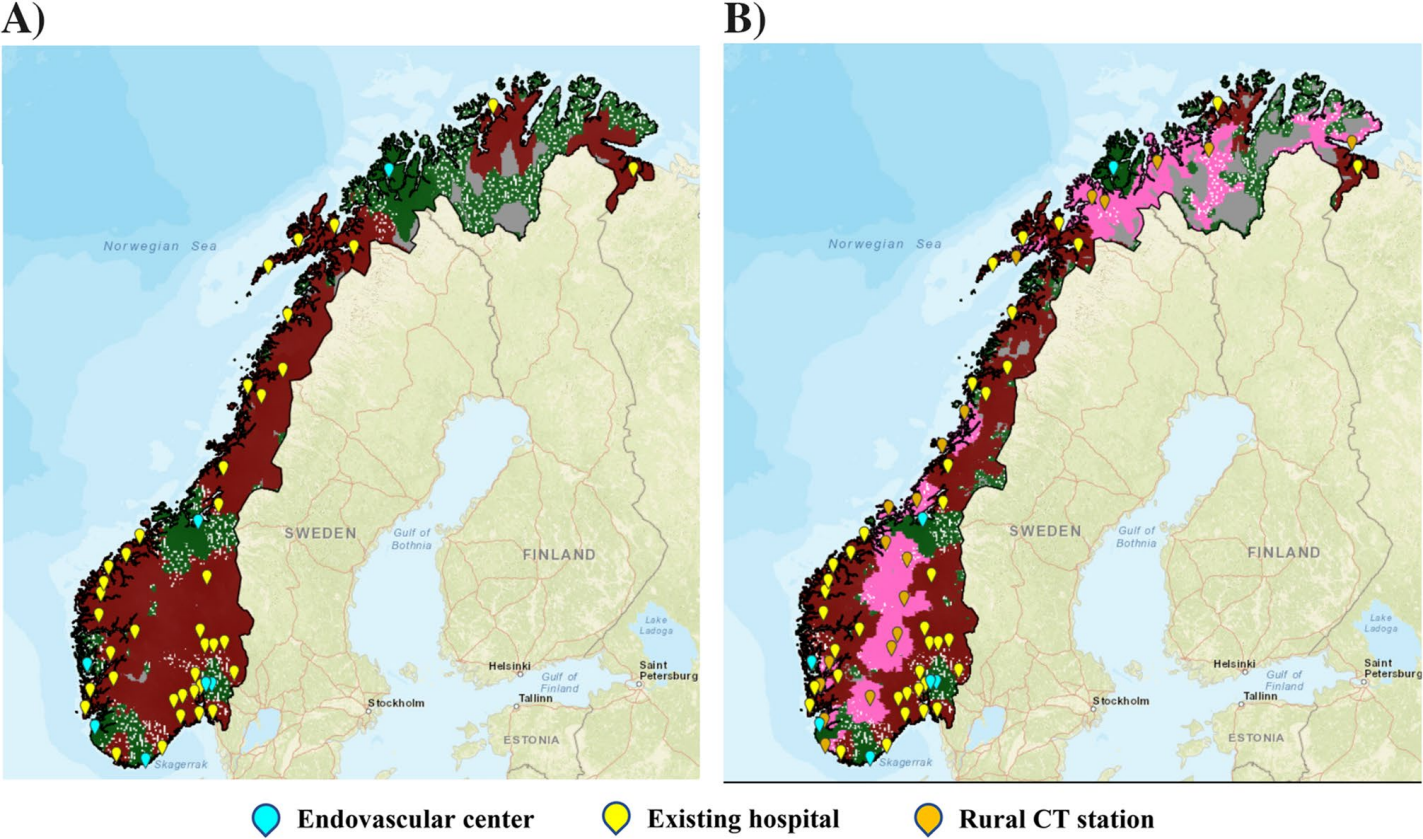
Transport routing of patients with suspected LVO stroke before (**A**) and after (**B**) deploying 20 rural CT stations. Green color depicts areas favouring mothership strategy – direct transport to EVT centre. Red color depicts areas which favour drip and ship via existing hospital. Pink color in **B**) depicts areas favouring drip and ship via rural CT station. Spotting of the colors illustrates probability of good outcome near equivalent regarding to the two types of transport strategy. Grey areas define areas with poor/no infrastructure excluding them from the model

Transport strategy for patients with suspected LVO stroke in the existing situation is illustrated in Fig. 4A. Prior to deployment of rural CT stations, the areas which favoured mothership transport were concentrated around the EVT centres. Areas which favoured drip and ship strategy were extensive and included 60% of the Norwegian geographical area. There were some spots amidst these rural areas so far from local hospitals that thrombolysis could not be administered within treatment time window and in this case mothership transport was preferred. The consequent changes in the strategy after hypothetical CT deployment is exemplified in Fig. 4B showing the results after placement of 20 CT stations. The deployment slightly increased the area of Norway in which patients with suspected LVO stroke should be transported according to a drip and ship strategy (via existing hospital or rural CT station) from 184,164 km^2^ to 193,657 km^2^ (5%). This corresponded to a decrease of mothership area from 25,647 km^2^ to 16,462 km^2^ (36%).

## Discussion

In this study we explored a solution to mitigate the transport delay for patients from rural areas with acute stroke by hypothetically deploying 10, 20 and 30 rural CT stations. By simulation, we markedly reduced OTT for the rural part of the population resulting in 87% potentially attaining thrombolysis within 180 min. The estimated mean OR for good outcome after thrombolysis for the rural population increased from 1.37 to 1.61. In cases of suspected LVO stroke, a 36% decrease in direct mothership transport areas was observed when deploying 30 CT stations.

In 2023, 22% of Norwegian patients with ischemic stroke received thrombolytic treatment and 6.4% were subject to thrombectomy [22]. 55% of all stroke patients were admitted to hospital later than four hours from onset of symptoms [22]. For rural patients with acute ischemic stroke, transport time constitutes a significant part of the delay to thrombolysis or thrombectomy. Consistent with our findings, an Australian study reported a lower rate of thrombolysis for patients living far from their stroke centre [14]. A U.S. study showed that rural stroke patients less frequently received intravenous thrombolysis or endovascular therapy and had higher in-hospital mortality than patients from urban areas [27].

In many countries, efforts are made to improve acute stroke treatment and optimize routing of patients between PSC and CSC. Recently, the Prehospital Stroke System of Care Consensus Conference in the U.S published specific recommendations for rural Stroke Systems of Care stressing prioritized efforts to ensure thrombolysis within 4.5 h from last known well for all eligible patients [28]. For patients with suspected LVO stroke, efficient transport to a thrombectomy-capable center should occur as soon as possible and in certain cases bypassing PSC. CTA for all patients at PSC is recommended, if needed interpreted by teleradiology.

A possible solution to tackle the transport time problem is the introduction of MSUs bringing CT examination and thrombolysis out to the patient. In Germany, MSUs has proven to reduce median time from alarm to therapy decision compared to in-hospital stroke treatment and prehospital intravenous thrombolysis for acute ischemic stroke has led to a better clinical outcome after three months in selected patients [29–31]. The MSU tested in Norway has recently proven to significantly reduce OTT and MSU patients were more often discharged home [32]. However, it is doubtful whether MSUs may be a viable solution in sparsely populated areas. An analysis from Norway concluded that 260 ischemic stroke patients would need to be treated annually with an MSU to achieve a reasonable cost-effectiveness compared to standard care [33].

An alternative to MSUs is rural CT stations which may increase the number of patients who will receive early stroke evaluation and conclusive treatment decisions. Rural CT stations may not be expected to provide definite decision criteria for the presence of LVO stroke at all hours. However, technical improvement has already made remotely controlled CTA available. In our clinical study, when CTA was absent, we considered patients with NIHSS > 6 with excluded cerebral haemorrhage as possible eligible for EVT. There is reason to believe that rapid local diagnostics and treatment lead to a more correct triage and more effective transport of patients, including the use of HEMS. Furthermore, rural CT stations may enable rapid anti-hypertensive treatment in individuals suffering from intracerebral hemorrhage [34]. Patients with major strokes benefitting from strict palliative care can be transferred directly to a local nursing home. Rural CT stations may be useful in trauma care, local diagnostics in conditions as confusion in the elderly and reduced consciousness of unknown cause. A rural CT station can perform outpatient controls for the local population. Finally, in contrast to MSUs, a rural CT station is more often functional, not occupied in transport. A simulation study in a rural area comparing local stationary CT, MSU and CT in HEMS has been conducted, and results encourage further development of prehospital CT models [35]. In this development, a stationary MSU could be one alternative to explore the potential for benefit of a rural CT station being able to move from location to location.

The inverse relationship between increasing time from onset of symptoms to treatment and decreasing OR for favourable outcome is continuous and exponential [2]. We divided our analysis in time slots of symptom onset to treatment of 180 and 270 min as set in the Norwegian Stroke Guidelines. Nevertheless, as modelled by Meretoja et al., every minute saved from ictus to revascularisation correspond to an average of at least 1.8 days of extra healthy life [36, 37]. Although benefitting relatively few patients, the deployment of 30 CT stations in the rural area led to a large gain in median transport time (74 min). The deployment of 10 and 20 CT stations seemed to linearly increase OR for good outcome, however the curve seemed to flatten out when 30 stations were deployed. As few as 10 CT stations decreased the estimated number of patients not eligible to thrombolytic therapy dramatically, partly alleviating the problem of unequal access to health care. A minimum of 10 CT stations were deployed due to the topography of Norway with large distances and poor road standard. In our simulation of 1,000 ischemic strokes, the calculated gain in transport time would, according to the model by Meretoja, correspond to approximately 135 days of extra healthy life for each patient with ischemic stroke in the rural subgroup.

There are several limitations to our study. Firstly, our initial clinical experience with a rural CT station remains to be expanded to a larger patient cohort. Secondly, we have not undertaken a cost benefit analysis of the rural CT concept. In a study published 2015, the net annual cost of the MSU in Berlin was €963,954 [38]. The authors concluded that this prehospital stroke concept had the potential for being health-economic reasonable with an incremental cost-effectiveness ratio of €32,456 per QALY. In comparison, our rural CT model in Vestre Viken Hospital Trust was established by investing approximately €465,000. The daily running costs are favourably influenced by having the service operated outside office hours by paramedical and hospital staff already on call. Thirdly, we based our analysis on at home stroke only and on the use of car ambulance alone, excluding the use of HEMS. It is likely that the use of HEMS is more frequent in rural areas, however less than 5% of Norwegian stroke patients are transported to hospital by HEMS (Norwegian Stroke Registry, unpublished). In addition, our simulation of 1000 strokes do not represent realistic real time data concerning stroke incident in rural areas, however, were performed for effect evaluation. Lastly, some components of time from onset of symptoms to treatment were estimated by us based on published data and may vary considerably among centres and regions.

In conclusion, this study presents a novel potentially viable solution for rapid stroke care in sparsely populated areas. Since most people live in city areas, such rural CT stations will serve a minor part of the population, but for this minority it might have a substantial effect on outcome after stroke. Provided our clinical experience so far is confirmed after further study, the implementation of this concept in other rural areas might be considered.

## Supplementary Information

Below is the link to the electronic supplementary material.

Supplementary Material 1

## Data Availability

The data that support the findings of this study are available from the Norwegian Mapping Authorities (NMA) and DESTINE Health but restrictions apply to the availability of these data, which were used under license for the current study, and so are not publicly available. Data are however, available from the authors upon reasonable request and with permission of NMA and DESTINE Health.

## References

[1] Wahlgren N, Ahmed N, Davalos A, Ford GA, Grond M, Hacke W, Hennerici MG, Kaste M, Kuelkens S, Larrue V, et al. Thrombolysis with alteplase for acute ischemic stroke in the Safe Implementation of Thrombolysis in Stroke-Monitoring Study (SITS-MOST): an observational study. Lancet. 2007;369:275–82. 10.1016/S0140-6736(07)60149-4. 17258667 10.1016/S0140-6736(07)60149-4

[2] Emberson J, Lees KR, Lyden P, Blackwell L, Albers G, Bluhmki E, Brott T, Cohen G, Davis S, Donnan G. Effect of treatment delay, age, and stroke severity on the effects of intravenous thrombolysis with alteplase for acute ischaemic stroke: a meta-analysis of individual patient data from randomised trials. Lancet. 2014;384(9958):1929–35. 10.1016/S0140-6736(14)60584-5. 25106063 10.1016/S0140-6736(14)60584-5 PMC4441266

[3] National Guidelines for Treatment and Rehabilitation of Stroke. National Directorate of Health. Hjerneslag - Helsedirektoratet. Accessed August. 2025.

[4] Wardlaw JM, Murray V, Berge E, del Zoppo GJ. Thrombolysis for acute ischaemic stroke. Cochrane Database of Systematic Reviews. Version published: 2014 July. 10.1002/14651858.CD000213.pub3 10.1002/14651858.CD000213.pub3 PMC4153726 25072528

[5] Norwegian Institute of Public Health. Effect of thrombolytic treatment 3 to 4.5 hours after onset of stroke. Effekt av trombolytisk behandling i intervallet 3 til 4,5 timer etter hjerneslag. 29553647

[6] Ma H, Campbell BCV, Parsons MW, Churilov L, Levi CR, Hsu C, Kleinig TJ, Wijeratne T, Curtze S, Dewey HM, et al. Thrombolysis Guided by Perfusion Imaging up to 9 Hours after Onset of Stroke. N Engl J Med. 2019;380:1795–803. 10.1056/nejmoa1813046. https://www.nejm.org/doi/full/. 31067369 10.1056/NEJMoa1813046

[7] Berkhemer OA, Fransen PS, Beumer D, Van den Berg LA, Lingsma HF, Yoo AJ, et al. A Randomized Trial of Intraarterial Treatment for Acute Ischemic Stroke. N Engl J Med. 2015;372:11–20. 10.1056/NEJMoa1411587. https://www.nejm.org/doi/full/. 25517348 10.1056/NEJMoa1411587

[8] Goyal M, Menon BK, van Zwam WH, Dippel DWJ, Mitchell PJ, Demchuk AM, et al. Endovascular thrombectomy after large-vessel ischaemic stroke: a meta-analysis of individual patient data from five randomised trials. Lancet. 2016;387:1723–31. 10.1016/S0140-6736(16)00163-X. 26898852 10.1016/S0140-6736(16)00163-X

[9] Nogueira RG, Jadhav AP, Haussen DC, Bonafe A, Budzik RF, Bhuva P, et al. Thrombectomy 6 to 24 Hours after Stroke with a Mismatch between Deficit and Infarct. N Engl J Med. 2018;378:11–21. 10.1056/NEJMoa1706442. https://www.nejm.org/doi/full/. 29129157 10.1056/NEJMoa1706442

[10] Albers GW, Marks MP, Kemp S, Christensen S, Tsai JP, Ortega-Gutierrez S, et al. Thrombectomy for Stroke at 6 to 16 Hours with Selection by Perfusion Imaging. N Engl J Med. 2018;378:708–18. 10.1056/NEJMoa1713973. https://www.nejm.org/doi/full/. 29364767 10.1056/NEJMoa1713973 PMC6590673

[11] Ospel JM, Goyal M. A review of endovascular treatment for medium vessel occlusion stroke. J NeuroInterventional Surg. 2021;13:623–30. 10.1136/neurintsurg-2021-017321. 10.1136/neurintsurg-2021-017321 33637570

[12] Norwegian Minstry of Health and Care Services. NOU Report 2015: 17. NOU 2015: 17 - regjeringen.no.

[13] Evenson KR, Foraker RE, Morris DL, Rosamond WD. A comprehensive review of prehospital and in-hospital delay times in acute stroke care. Int J Stroke. 2009;4:187–99. 10.1111/j.1747-4949.2009.00276.x. 19659821 10.1111/j.1747-4949.2009.00276.x PMC2825147

[14] Leyden JM, Chong WK, Kleinig T, Lee A, Field JB, Jannes J. A population-based study of thrombolysis for acute stroke in South Australia. Med J Aust. 2011;194:111–5. 10.5694/j.1326-5377.2011.tb04191.x. 21299483 10.5694/j.1326-5377.2011.tb04191.x

[15] Ranhoff Hov M, Zakariassen E, Lindner T, Nome T, Bache KG, Røislien J, et al. Interpretation of Brain CT Scans in the Field by Critical Care Physicians in a Mobile Stroke Unit. J Neuroimaging. 2018;28:106–11. 10.1111/jon.12458. 28766306 10.1111/jon.12458 PMC5811888

[16] Ranhoff Hov M, Nome T, Zakariassen E, Russell D, Røislien J, Lossius HM, et al. Assessment of acute stroke cerebral CT examinations by anaesthesiologists. Acta Anaesthesiol Scand. 2015;59:1179–86. 10.1111/aas.12542. 25976840 10.1111/aas.12542 PMC5029598

[17] Ranhoff Hov M, Røislien J, Lindner T, Zakariassen E, Bache KC, Solyga VM, et al. Stroke severity quantification by critical care physicians in a mobile stroke unit. Eur J Emerg Med. 2017;26:194–8. 10.1097/mej.0000000000000529. 10.1097/MEJ.0000000000000529 PMC6504122 29239899

[18] Ibsen J, Ranhoff Hov M, Tokerud GE, Fuglum J, Linnerud Krogstad M, Stugaard M, et al. Prehospital Computed Tomography in a rural district for rapid diagnosis and treatment of stroke. Eur Stroke J. 2024;0(0). 10.1177/23969873241267084. 10.1177/23969873241267084 PMC11556544 39340436

[19] Norwegian Ministry of Transport and Communications. National Transport Plan 2018–2029. Meld. St. 33 (2016–2017). Accessed August 2025.

[20] The National Road Database. The Norwegian Public Road administration 2018. https://www.regjeringen.no/en/dep/sd/organisation/subordinate-agencies-and-enterprises/norwegian-public-roads-administration/id443412/. Accessed July 2025.

[21] Statistics Norway. Population – Key Figs. 2023. https://www.ssb.no/befolkning/statistikker/beftett. Accessed August 2024.

[22] The Norwegian Stroke Registry. Annual Report 2023. Norwegian Medical Quality Registry. https://www.kvalitetsregistre.no/sites/default/files/2024-06/Årsrapport%202023%20Norsk%20hjerneslagregister.pdf

[23] National Health Quality Indicators. The Norwegian Directorate of Health 2018. National Healthcare Quality Indicators - Helsedirektoratet. Accessed August 2025.

[24] Drenck N, Viereck S, Stokholm Bækgaard J, Bang Christensen K, Lippert F, Folke F. Pre-hospital management of acute stroke patients eligible for thrombolysis – an evaluation of ambulance on-scene time. Scand J Trauma Resusc Emerg Med. 2019;27:3. 10.1186/s13049-018-0580-4. 30626404 10.1186/s13049-018-0580-4 PMC6327613

[25] Freyssenge J, Renard F, Schott AM, Derex L, Nighoghossian N, Tazaroutre K, El Khoury C. Measurement of the potential geographic accessibility from call to definitive care for patient with acute stroke. Int J Health Geogr. 2018;17:1. 10.1186/s12942-018-0121-4. 29329535 10.1186/s12942-018-0121-4 PMC5767021

[26] Holodinsky JK, Williamson TS, Demchuk AM, Zhao H, Zhu L, Francis MJ, et al. Modeling Stroke Patient Transport for All Patients With Suspected Large-Vessel Occlusion. JAMA Neurol. 2018;75:1477–86. 10.1001/jamaneurol.2018.2424. 30193366 10.1001/jamaneurol.2018.2424 PMC6583214

[27] Hammond G, Luke AA, Elson L, Towfighi A, Joynt Maddox KE. Urban-rural inequities in acute stroke care and in-hospital mortality. stroke. 2020;51:2131–8. 10.1161/STROKEAHA.120.029318. 32833593 10.1161/STROKEAHA.120.029318

[28] Jauch EC, Schwamm LH, Panagos PD, Barbazzeni J, Dickson R, Dunne R, et al. Recommendations for Regional Stroke Destination Plans in Rural, Suburban, and Urban Communities From the Prehospital Stroke System of Care Consensus Conference. Stroke. 2021;52:e133-e152. 10.1161/strokeaha.120.033228 10.1161/STROKEAHA.120.033228 33691507

[29] Fassbender K, Grotta JC, Walter S, Grunwald IQ, Ragoschke-Schumm A, Saver JL. Mobile stroke units for prehospital thrombolysis, triage, and beyond: benefits and challenges. Lancet. 2017;16:227–37. 10.1016/s1474-4422(17)30008-x. 10.1016/S1474-4422(17)30008-X 28229894

[30] Walter S, Kostopoulos P, Haass A, Keller I, Lesmeister M, Schlechtriemen T, et al. Diagnosis and treatment of patients with stroke in a mobile stroke unit versus in hospital: a randomised controlled trial. Lancet Neurol. 2012;11:397–404. 10.1016/s1474-4422(12)70057-1. 22497929 10.1016/S1474-4422(12)70057-1

[31] Nolte CH, Ebinger M, Scheitz JF, Kunz A, Erdur H, Geisler F, et al. Effects of Prehospital Thrombolysis in Stroke Patients With Prestroke Dependency. Stroke. 2018;49:646–51. 10.1161/strokeaha.117.019060. 29459395 10.1161/STROKEAHA.117.019060

[32] Larsen K, Jæger HS, Tveit LH, Ranhoff Hov M, Thorsen K, Røislien J, et al. Ultraearly Thrombolysis by an Anesthesiologist in a Mobile Stroke Unit. A prospective, controlled intervention study. Eur J Neurol. 2021. 10.1111/ene.14877. 33890385 10.1111/ene.14877

[33] Lund UH, Stoinska-Scheider A, Larsen K, Bache KG, Robberstad B. Cost-effectiveness of Mobile Stroke Unit Care in Norway. Stroke. 2022;53(10):3173–81. 10.1161/STROKEAHA.121.037491. 35862205 10.1161/STROKEAHA.121.037491 PMC9508956

[34] Ma L, Hu X, Song L, Chen X, Ouya M, Billot L, et al. The third Intensive Care Bundle with Blood Pressure Reduction in Acute Cerebral Haemorrhage Trial (INTERACT3): an international, stepped wedge cluster randomised controlled trial. Lancet July. 2023;01(10395):27–40. 10.1016/S0140-6736(23)00806-1. 10.1016/S0140-6736(23)00806-1 PMC10401723 37245517

[35] Christensen E, Fagerheim Bugge H, Hagemo J, Larsen K, Harring AKV, Gleditsch J, et al. Prehospital stroke diagnostics using three different simulation methods: A pragmatic pilot study. Eur Stroke J. 2024;0(0). 10.1177/23969873241252564. 10.1177/23969873241252564 PMC11569525 38751332

[36] Meretoja A, Keshtkaran M, Saver JL, Tatlisumak T, Parsons MW, Kaste M, et al. Stroke thrombolysis: save a minute, save a day. Stroke. 2014;45:1053–8. 10.1161/strokeaha.113.002910. 24627114 10.1161/STROKEAHA.113.002910

[37] Meretoja A, Keshtkaran M, Tatlisumak T, Donnan GA, Churilov L. Endovascular therapy for ischemic stroke: Save a minute-save a week. Neurology. 2017;88:2123–7. 10.1212/WNL.0000000000003981. 28455382 10.1212/WNL.0000000000003981

[38] Gyrd-Hansen D, Olsen KR, Bollweg K, Kronborg C, Ebinger M, Audebert HJ. Cost-effectiveness estimate of prehospital thrombolysis: results of the PHANTOM-S study. Neurology. 2015;84:1090–7. 10.1212/wnl.0000000000001366. 25672925 10.1212/WNL.0000000000001366

