# Supplementary material for: Rural Computed Tomography - a model for access to rapid stroke care in sparsely populated areas?
Source: BMC Health Serv Res. 2026 Mar 19;26:596. doi: 10.1186/s12913-026-14307-6 (PMC13123209; doi:10.1186/s12913-026-14307-6)
Supplement: Supplementary file 1 — Supplementary Material 1 [file 12913_2026_14307_MOESM1_ESM.docx]

**Supplementary Materials**

**Supplementary Table 1**

| **Table 1. Estimated median time components from onset of symptoms to treatment for the rural subgroup in the existing situation and after deployment of CT stations** | | | | | | | | | |
| --- | --- | --- | --- | --- | --- | --- | --- | --- | --- |
| Onset of symptoms to alarm, minutes (IQR)^1^ | | | | | |  |  | **Existing**  34 (25-40) | **10 CT 20 CT 30 CT**  34 (25-40) 34 (25-40) 34 (25-40) |
| Alarm to arrival of ambulance, minutes (IQR)^2^ |  |  | | | | | | 25 (18-35) | 25 (18-35) 25 (18-35) 25 (18-35) |
| Ambulance on scene time, minutes (IQR)^3^  Transport time, minutes (IQR)^4^  Door to needle time, minutes (IQR)^5^ | | |  |  | 21 (16-27)  105 (83-135)  30 (25-38) | | | | 21 (16-27) 21 (16-27) 21 (16-27)  74 (31-105) 48 (15-82) 31 (9-72)  30 (25-38) 30 (25-38) 30 (25-38) |
| Estimated onset of symptoms to treatment  ^1,5^ Norwegian Stroke Registry (22)  ^2^ Norwegian Health Quality Data Base (23)  ^3^ Data from a Danish prehospital study (24)  ^4^ Median transport time in ambulance | | |  |  | 215 | | | | 184 158 141 |

**Supplementary Table 2**

| **Table 2. Gamma distribution to model activity duration** |  |
| --- | --- |
| **Activity duration** | **Parameters** |
| Ictus to alarm | shape α = 11.56 ; scale β = 2.94 |
| Alarm to arrival of ambulance | shape α = 6.25 ; scale β = 4 |
| Ambulance on scene | shape α = 4.41 ; scale β = 4.76 |
| Transport time – rural subgroup: 0 CT | shape α = 6.89 ; scale β = 15.24 |
| Transport time – rural subgroup: 10 CT | shape α = 6.08 ; scale β = 12.16 |
| Transport time – rural subgroup:2 0 CT | shape α = 2.56 ; scale β = 18.75 |
| Transport time – rural subgroup: 30 CT | shape α = 1.07 ; scale β = 29.03 |
| Door to needle time | shape α = 9; scale β = 3.33 |


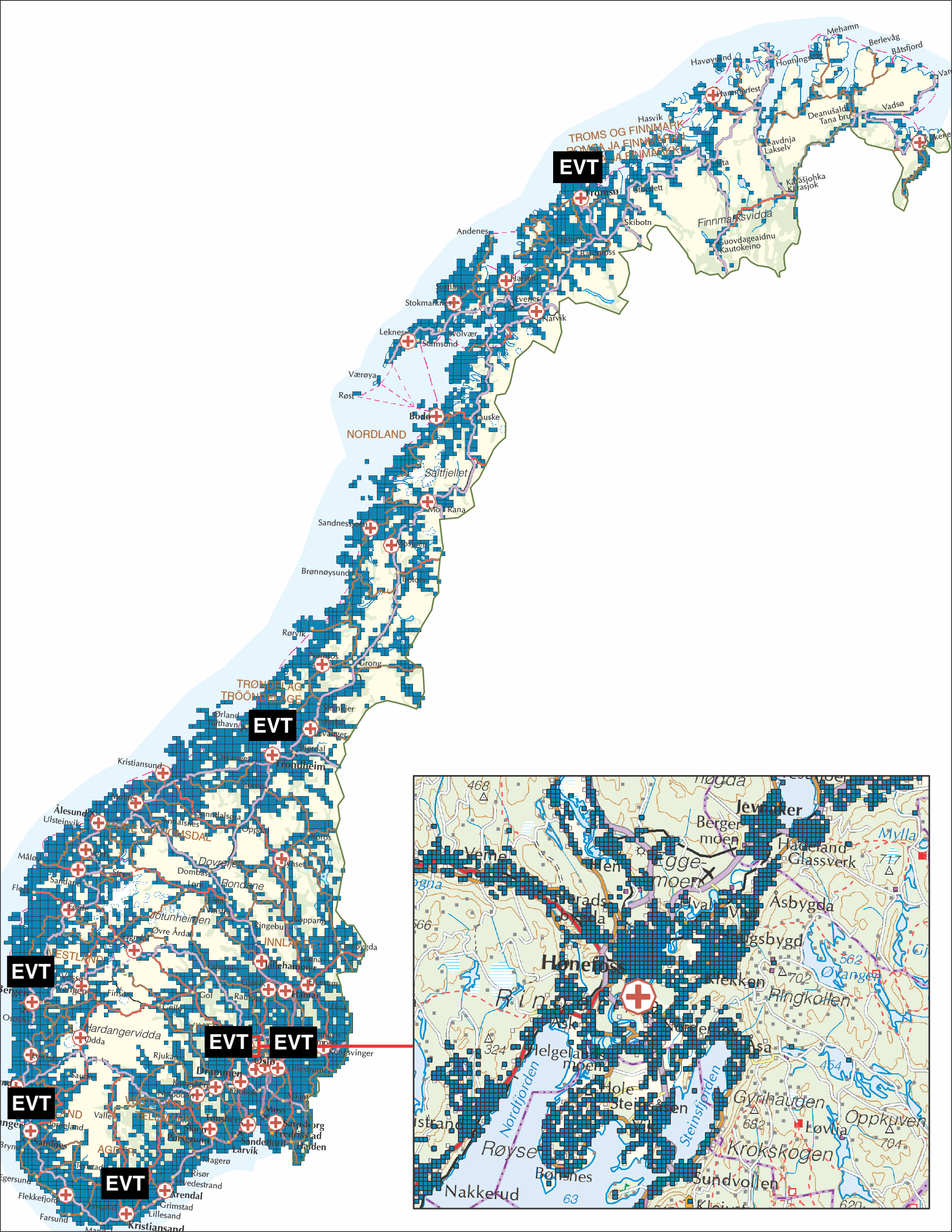


**Supplementary Figure 1.** Norway divided in populated geographical polygons of 250 m^2^. Red crosses represent the 48 hospitals in which acute stroke patients are admitted. EVT: Comprehensive Stroke Centres providing Endovascular Treatment


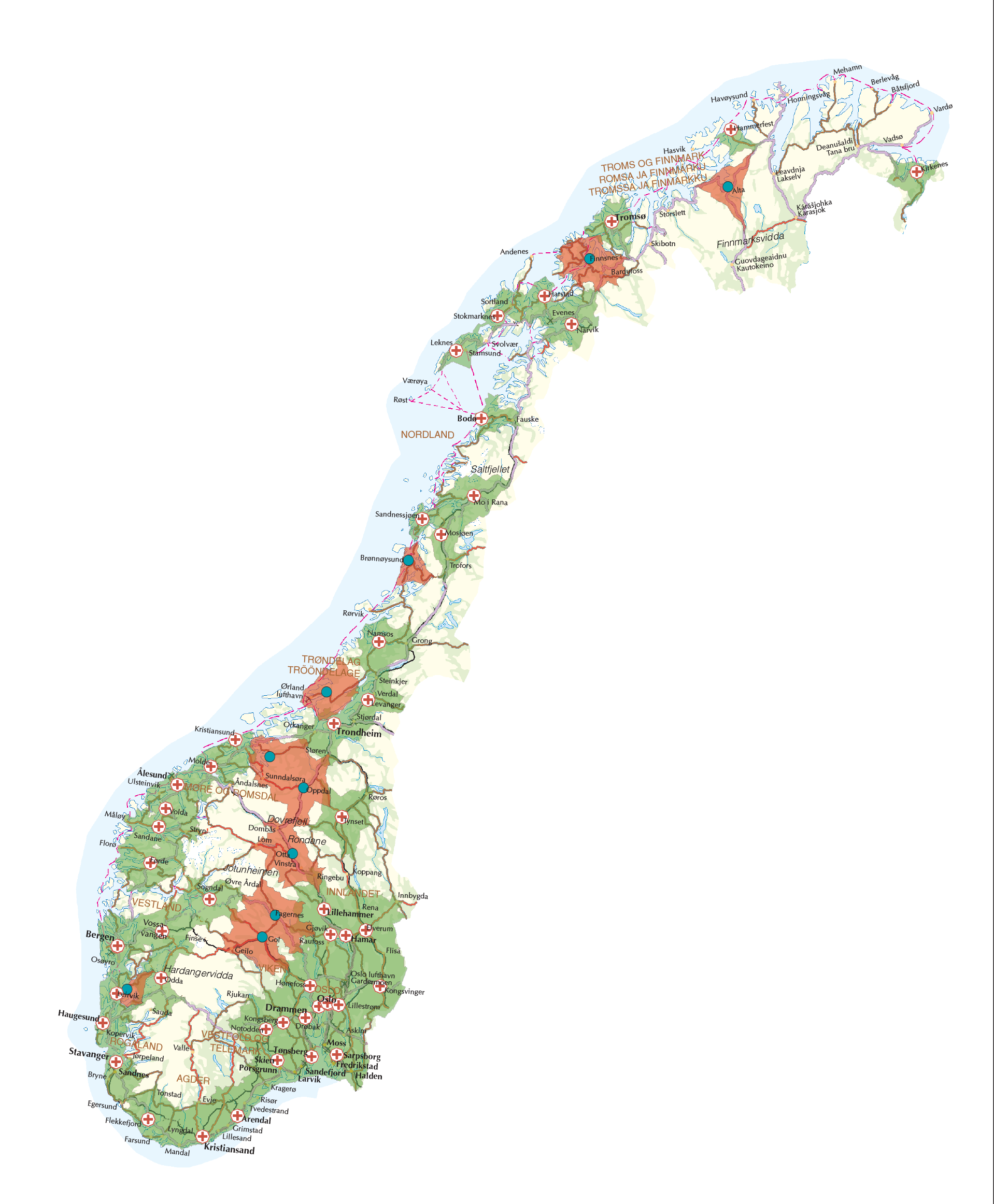


**Supplementary Figure 2.** The deployment of 10 CT stations for the rural subgroup. Green color depicts areas in which the population live < 70 minutes of ambulance transport time from an existing hospital. Red color depicts catchment areas for the deployed CT stations with ambulance transport time < 70 minutes.


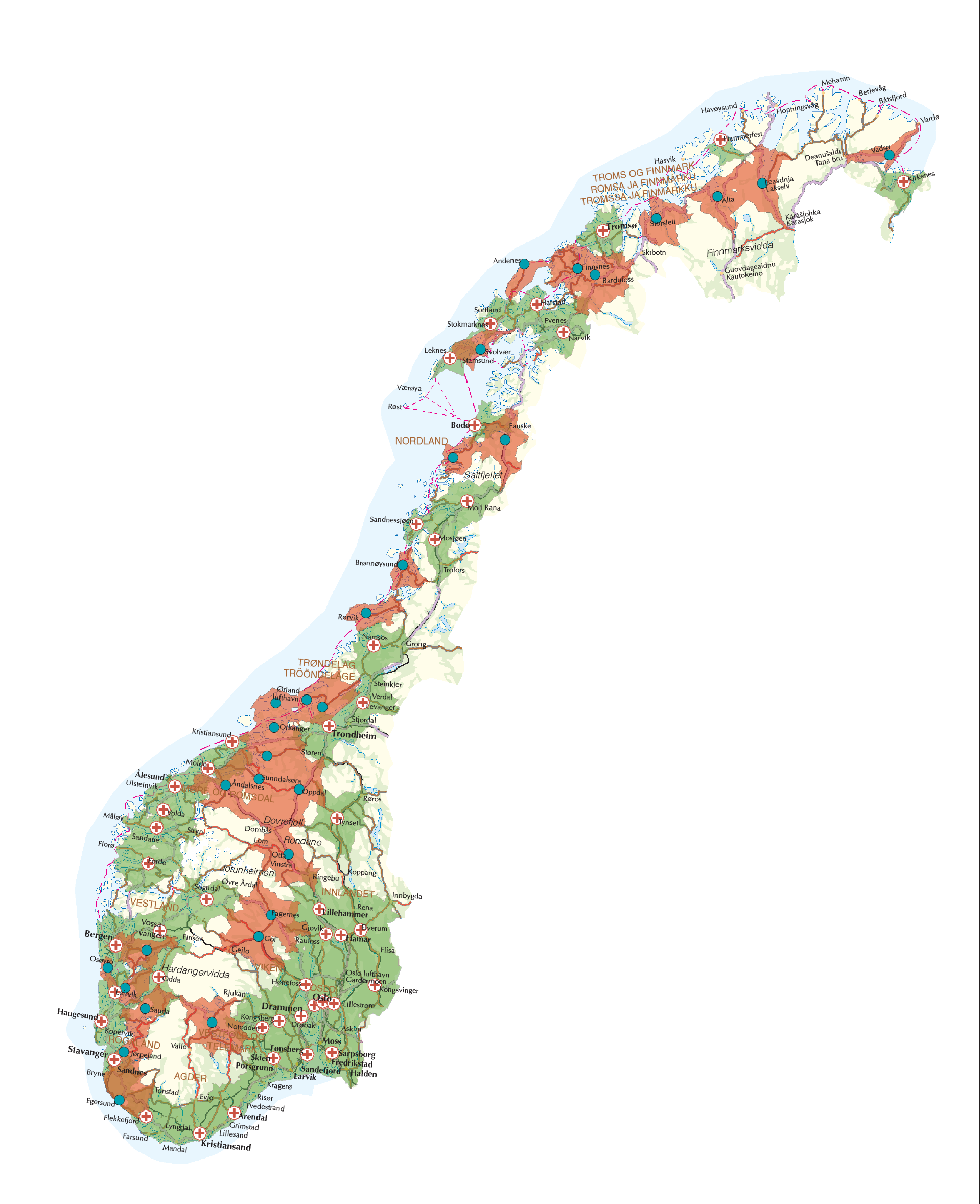


**Supplementary Figure 3.** The deployment of 30 CT stations for the rural subgroup. Green color depicts areas in which the population live < 70 minutes of ambulance transport time from an existing hospital. Red color depicts catchment areas for the deployed CT stations with ambulance transport time < 70 minutes.
